# Transform-limited single photons from a single quantum dot

**DOI:** 10.1038/ncomms9204

**Published:** 2015-09-08

**Authors:** Andreas V. Kuhlmann, Jonathan H. Prechtel, Julien Houel, Arne Ludwig, Dirk Reuter, Andreas D. Wieck, Richard J. Warburton

**Affiliations:** 1 Department of Physics, University of Basel, Klingelbergstrasse 82, CH-4056 Basel, Switzerland; 2 Institut Lumière Matière (ILM), UMR5306 Université Lyon 1/CNRS, Université de Lyon, 69622 Villeurbanne Cedex, France; 3 Lehrstuhl für Angewandte Festkörperphysik, Ruhr-Universität Bochum, D-44780 Bochum, Germany; 4 Department Physik, Universität Paderborn, Warburger Strasse 100, D-33098 Paderborn, Germany

## Abstract

Developing a quantum photonics network requires a source of very-high-fidelity single photons. An outstanding challenge is to produce a transform-limited single-photon emitter to guarantee that single photons emitted far apart in the time domain are truly indistinguishable. This is particularly difficult in the solid-state as the complex environment is the source of noise over a wide bandwidth. A quantum dot is a robust, fast, bright and narrow-linewidth emitter of single photons; layer-by-layer growth and subsequent nano-fabrication allow the electronic and photonic states to be engineered. This represents a set of features not shared by any other emitter but transform-limited linewidths have been elusive. Here, we report transform-limited linewidths measured on second timescales, primarily on the neutral exciton but also on the charged exciton close to saturation. The key feature is control of the nuclear spins, which dominate the exciton dephasing via the Overhauser field.

## Abstract (web summary)

Photons emitted from a quantum dot typically have slightly different frequencies owing to various sources of noise. Here, the authors suppress the noise, notably the noise arising from the nuclear spins, and demonstrate single-photon emission with a transform-limited optical linewidth.

## Graphical abstract

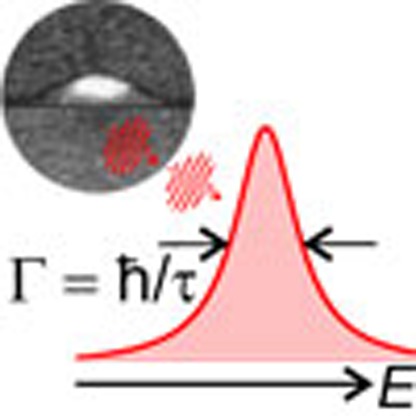

A key goal in quantum communication is to create highly indistinguishable photons that are separated in space by more than 100 km for device-independent quantum key distribution and for a quantum repeater^1^. This is potentially possible using a solid-state source, a semiconductor quantum dot. A single quantum dot mimics a two-level atom and single photons are generated either by spontaneous emission from the upper level^2^,^3^,^4^ or by coherent scattering of a resonant laser^5^,^6^,^7^. The radiative lifetime is typically *τ*_r_=800 ps (ref. 8). There is evidence that on this timescale and at low temperature, there is negligible pure upper level decoherence^5^,^6^,^7^,^9^. Photons emitted subsequently are close to indistinguishable^3^,^10^. (At higher temperatures^9^,^11^,^12^,^13^, equivalently at low temperature but at high Rabi couplings^14^,^15^, phonons dephase the upper level.) A key remaining issue concerns the wandering of the centre frequency over times much longer than *τ*_r_ (refs 16, 17, 18). This wandering is highly problematic in any quantum photonics network: the quantum dot detunes from the common optical frequency and becomes dark; equivalently, the indistinguishability of quantum dot single photons generated far apart in the time domain is reduced. Active single-quantum-dot stabilization is possible but is presently limited to correcting for very slow drifts and in any case comes at the expense of complexity^18^,^19^. Eliminating the spectral wanderings would be highly advantageous.

The spectral wanderings can be conveniently probed simply by measuring the optical linewidth. Measured on millisecond or even second timescales, the quantum dot optical linewidth Γ is larger than the transform limit Γ_0_=*ℏ*/*τ*_r_ (refs 16, 17, 20, 21). In fact single-quantum-dot linewidths have remained stubbornly 50–100% above the transform limit even under the most favourable conditions (high-quality material, low temperature, charge control via Coulomb blockade and resonant excitation). We report here two regimes in which we observe transform-limited quantum dot optical linewidths even when measured on second timescales. One regime applies to the neutral exciton, X^0^, the other to the charged exception, X^1−^.

The X^0^ transition is split into two linearly polarized transitions by the electron–hole exchange, the so-called fine structure, corresponding to an admixture of the spin ±1 states (Fig. 1a). The splitting between the two transitions increases in an applied magnetic field, quadratically initially (Fig. 1c). The magnetic field is applied externally or it arises from a net polarization in the nuclear spins, which acts on the electron spin via the Overhauser field, *B*_N_. The X^1−^ exhibits a single line at zero magnetic field (Fig. 1b) splitting linearly in magnetic field, again via an external field or Overhauser field (Fig. 1d). Both excitons exhibit large and similar d.c. Stark shifts (dependence of energy on electric field *F*), ∼25 μeV cm kV^−1^ (ref. 22). Charge noise leads to an inhomogeneous broadening of both X^0^ and X^1−^ transitions via the d.c. Stark shift. This determines the inhomogeneous broadening for quantum dots in poor-quality material or quantum dots in high-quality material but with non-resonant excitation. In addition, both excitons are sensitive to spin noise, that is, fluctuations in the Overhauser field, but with different sensitivities. For X^0^, the sensitivity is second order as the hole ‘shields' the electron from the spin noise; for X^1−^ the sensitivity is first order on account of the unpaired electron in the X^1−^ ground state. For instance, a typical Overhauser field of 20 mT (ref. 23) (arising from incomplete cancellation of the ∼10^5^ nuclear spins^24^,^25^) leads to a linewidth contribution in the case of X^1−^ of ∼0.5–1.0 μeV. Experimentally, there is strong evidence that in this cold, clean limit, spin noise and not charge noise is responsible for the X^1−^ inhomogeneous broadening^18^,^21^. Despite the different sensitivity to spin noise the X^0^ and X^1−^ linewidths are very similar^16^,^17^,^21^.

The approach here is to suppress the effects of charge noise by working in the ideal limit (high-quality material at low temperature, resonant excitation on a quantum dot in the Coulomb blockade regime), to compare X^0^ and X^1−^ on the same quantum dot and to suppress the effects of spin noise by a search of the available parameter space. The improvement in the optical linewidth arises as a consequence of improved optical control of the nuclear spins associated with the quantum dot. It is noteworthy that the hyperfine interaction^26^,^27^ limits also the entanglement in the biexciton cascade^28^,^29^.

## Results

### X^0^ single-quantum-dot optical linewidth

**Figure 2:**
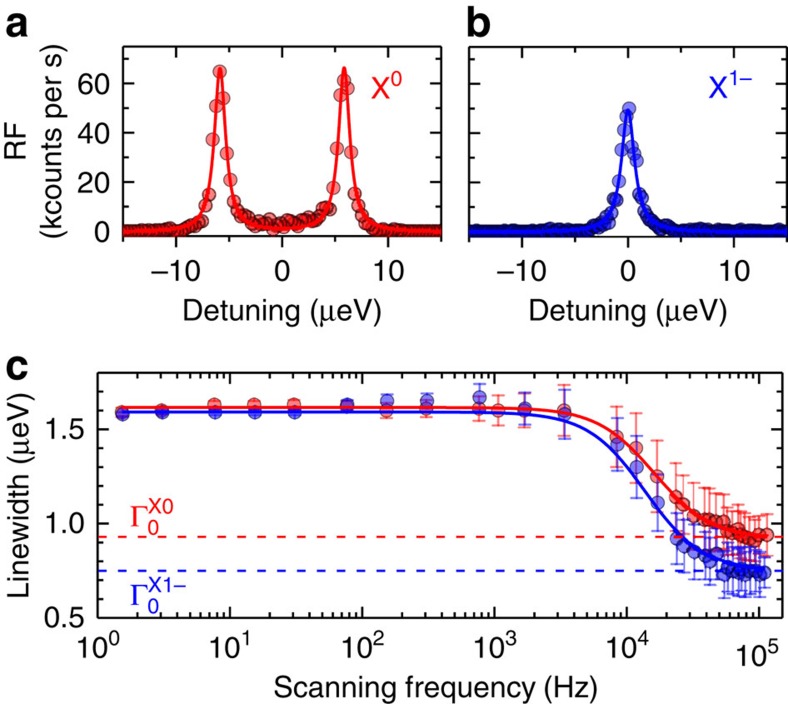
Linewidth versus scanning frequency. (**a**,**b**) X^0^, X^1−^ resonance fluorescence versus detuning *δ* at 4.2 K, *B*=0.0 mT with 100 ms integration time per point. The solid lines are Lorentzian fits to the data. The linewidths are 
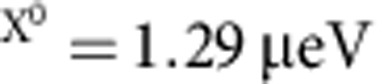
, 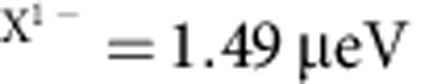; the Rabi energies Ω/Γ_0_=0.5 (X^0^), 0.4 (X^1−^); and transform limits 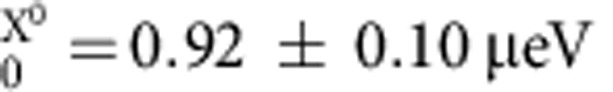, 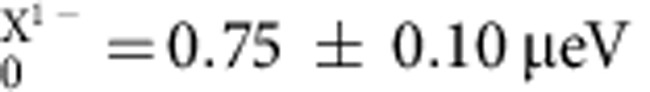. (**c**) RF linewidth against scanning frequency d*δ*/d*t*/Γ_0_. Γ approaches Γ_0_ for scanning frequencies above 50 kHz. For each scanning frequency, the error bar represents the s.d. of several hundred linewidth scans. Solid lines represent a Lorentzian fit of the data with linewidth 30±3 kHz.

A typical X^0^ resonance fluorescence (RF) spectrum is shown in Fig. 2a with Ω/Γ_0_=0.5 where Ω is the Rabi coupling. The linewidth is a factor of 1.4 larger than the transform limit (for this particular quantum dot, 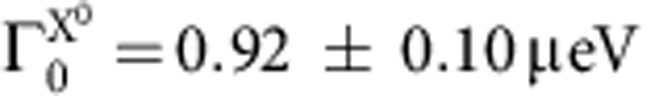). The transform limit Γ_0_ is measured by scanning the optical resonance very quickly such that the fluctuations are frozen during the measurement^21^ (Fig. 2c). The result is corroborated by measuring the radiative lifetime, either by recording a decay curve following pulsed excitation or by recording an intensity correlation *g*^(2)^: the results agree to within the random errors of ∼5% (Supplementary Note 1 and Supplementary Fig. 1).

**Figure 3:**
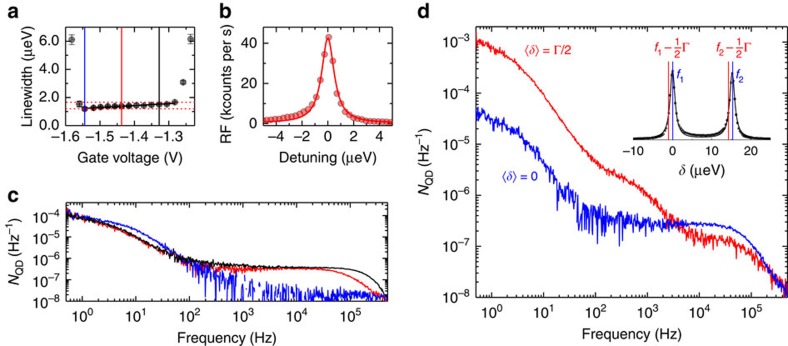
X^0^ spin noise within the Coulomb blockade plateau. (**a**) X^0^ optical linewidth measured at Rabi energies Ω/Γ_0_=0.50 for different gate voltages by sweeping the laser frequency through the resonance and integrating 100 ms per point. Γ decreases from 1.66 to 1.19 μeV with decreasing gate voltage. (**b**) X^0^ spectrum with Γ=1.15 μeV at *V*_g_=−1.54 V. (**c**) X^0^ noise spectra recorded at Rabi energies Ω/Γ_0_=0.65 for different voltages, indicated in **a** by solid lines. Maximum/minimum spin noise (black/blue) is correlated with the largest/smallest Γ. (**d**) *N*_QD_(*f*) on X^0^ recorded with two lasers of frequencies *f*_1_ and *f*_2_ and a frequency splitting *f*_1_−*f*_2_ equal to the fine structure splitting for 〈*δ*〉=0 (blue) and 〈*δ*〉=Γ/2 (red). Inset shows the laser frequency detuning relative to the optical resonance.

Figure 3a shows Γ versus *V*_g_ on the neutral exciton, X^0^, measured below but close to saturation, Ω/Γ_0_=0.5. At the edges of the Coulomb blockade plateau, Γ rises rapidly on account of fast electron spin dephasing via co-tunnelling with the Fermi sea^30^. This process slows down as *V*_g_ moves away from the plateau edges. The new feature is that a ‘sweet spot' exists close to the negative *V*_g_ end of the plateau with minimum linewidth 1.19±0.13 μeV (Fig. 3a,b). Accounting for the small power broadening, the ideal limit is 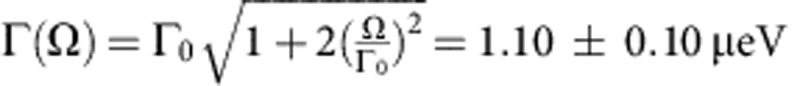. Within the measurement uncertainties of 10%, the transform limit is therefore achieved. As *V*_g_ raised to the positive side of the sweet spot, Γ increases beyond the ideal limit (Fig. 3a).

It is instructive to investigate the sources of noise. A diagnostic is a noise spectrum *N*_QD_(*f*), a Fourier transform of the RF time-trace (Supplementary Note 2 and Supplementary Fig. 2). From the known relationships between RF signal, detuning *δ*, Rabi coupling Ω, electric field *F* and the Overhauser field *B*_N_, the variances *F*_r.m.s._ and *B*_N,r.m.s._ can be determined from the noise spectrum^21^ (Supplementary Note 3). The increase in linewidth above the transform limit represents a sum over all noise sources from the scanning frequency, about 1 Hz, to Γ_0_/*ℏ*, about 1 GHz. The noise spectra at the low-bias end (the sweet spot), the centre of the plateau and the positive-bias end are shown in Fig. 3c. There is a Lorentzian feature with linewidth 30 Hz (noise correlation time 30 ms) and a second Lorentzian feature at higher frequencies with linewidth 200 kHz (correlation time 5 μs). The origin of the two features in the noise spectrum can be identified by exploiting the different X^0^ response to charge noise and spin noise: charge noise moves both X^0^ peaks rigidly together; spin noise moves them apart or closer together, a ‘breathing' motion. A two-laser experiment enables us to distinguish between these two possibilities. Specifically, we record X^0^ noise spectra with two lasers with frequencies separated in frequency by the fine structure. On detuning both lasers from *δ*=0 to *δ*=Γ/2, the sensitivity to charge noise increases (changing from second order to first order) yet the sensitivity to spin noise decreases (remaining second order but with a reduced pre-factor) (Supplementary Note 4 and Supplementary Fig. 3). In the experiment, switching from 〈*δ*〉=0 to 〈*δ*〉=Γ/2 causes the noise power of the low-frequency component to increase markedly (Fig. 3d) identifying it as charge noise. However, the frequency sum over the charge noise gives a contribution to Γ smaller than 0.05 μeV (Supplementary Note 5), a negligible value. (We note that both the d.c. Stark coefficient and Γ vary from quantum dot to quantum dot yet there is no correlation between the two (Supplementary Note 6 and Supplementary Fig. 4), pointing also to the unimportance of charge noise in the optical linewidth.) Conversely, the noise power of the high-frequency component decreases on detuning both lasers from *δ*=0 to *δ*=Γ/2, identifying it as spin noise (Fig. 3d). Furthermore, noise spectra measured at 〈*δ*〉=0 but with a single laser tuned to one of the X^0^ transitions show that the low-frequency noise, the charge noise, is similar for all three biases yet the high-frequency noise, the spin noise, increases with increasing bias (Fig. 3c). This confirms that the high-frequency noise, the spin noise, is responsible for the inhomogeneous linewidth: the integrated spin noise is vanishingly small at the sweet spot, increasing at the centre of the plateau, and increasing further at the positive-bias edge.

**Figure 4:**
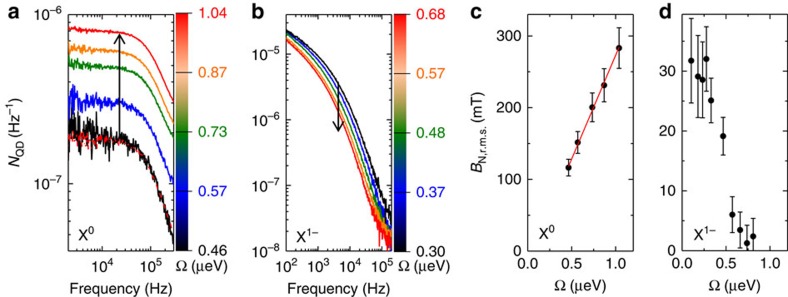
Spin noise power dependence (**a**) *N*_QD_(*f*) on X^0^ for a series of Rabi couplings Ω with *V*_g_ at the centre of the Coulomb blockade plateau at *B*=10.0 mT. The experimental data for Ω=0.46 μeV (black curve) is accompanied by the Monte Carlo fit (red dashed curve). (**b**) *N*_QD_(*f*) on X^1−^ for a series of Rabi couplings Ω taken at *B*=10.0 mT to enhance the sensitivity to spin noise (Supplementary Note 8). (**c**,**d**) *B*_N,r.m.s._ versus Ω for X^0^, X^1−^. For each Rabi coupling, the error bar in **c** represents an uncertainty of 10% on the determination of *B*_N,r.m.s._ from the Monte Carlo fit; the error bar in **d** represents the s.d. of several scans. r.m.s, root mean squared.

In general, the X^0^ Γ versus Ω curve does not follow exactly the textbook result for a two-level system (Supplementary Note 7 and Supplementary Fig. 5). The Ω dependence of *N*_QD_(*f*) is highly revealing (Fig. 4a, Supplementary Note 8 and Supplementary Fig. 6). In the centre of the plateau, as Ω increases the X^0^ spin noise also increases (Fig. 4a). 
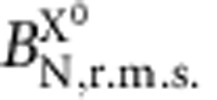
 increases roughly linearly with Ω reaching at the highest couplings extremely high values, 300 mT (Fig. 4c). (
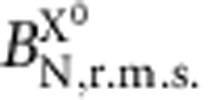
 is determined by a Monte Carlo simulation of *N*_QD_(*f*) including an ensemble of fluctuating nuclei—this is robust as X^0^ is sensitive only to the vertical component of *B*_N_ (Supplementary Note 3).) The large 
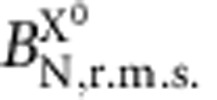
 would appear to prohibit transform-limited linewidths on X^0^ at all but the very lowest optical couplings. However, at the sweet spot, this mechanism is clearly suppressed: 
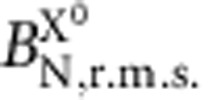
 reduces to <50 mT and approaches the value for a quantum dot in the ground state.

The existence of the X^0^ sweet spot is a robust phenomenon. It exists on all the quantum dots investigated in this particular sample, on quantum dots from other samples from the same wafer and from samples from other wafers of a similar but non-identical design (Supplementary Note 9 and Supplementary Fig. 7). A very striking example is the observation of the sweet spot on a p-type field-effect device (Supplementary Fig. 7(e)). Choosing the correct bias allows us to achieve X^0^ transform-limited lifetimes (to within the random error of 0.1 μeV) in each case.

### X^1−^ single-quantum-dot optical linewidth

A typical X^1−^ resonance fluorescence spectrum is shown in Fig. 2b with Ω/Γ_0_=0.4 (same quantum dot as in Fig. 2a). The linewidth is a factor of 2.0 larger than the transform limit (for this particular quantum dot, 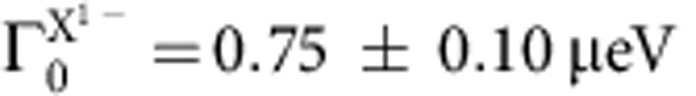). For X^1−^, it is clear that the nuclear spins are a significant source of inhomogeneous broadening. As a function of bias, the X^1−^ linewidth is smallest in the centre of the Coulomb blockade plateau, rising at the edges. This is consistent with a co-tunnelling dominated mechanism^30^ (Supplementary Note 9 and Supplementary Fig. 7(a)). We investigate the spin noise and in particular its Ω dependence via the noise spectra. Figure 4b shows that the X^1−^ spin noise decreases as Ω increases, corresponding to a decrease in 
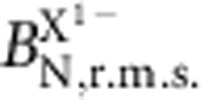
 (Fig. 4d). (The distinction between charge noise and spin noise can be made in the case of X^1−^ simply by changing the detuning from 〈*δ*〉=0 to 〈*δ*〉=Γ/2 in a one-laser experiment^21^. X^1−^ responds to all three components of *B*_N_, a more complex problem than that for X^0^, and instead 
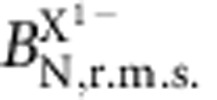
 is determined (Fig. 4d) with lower systematic error from the two-laser experiment described below.)

**Figure 5:**
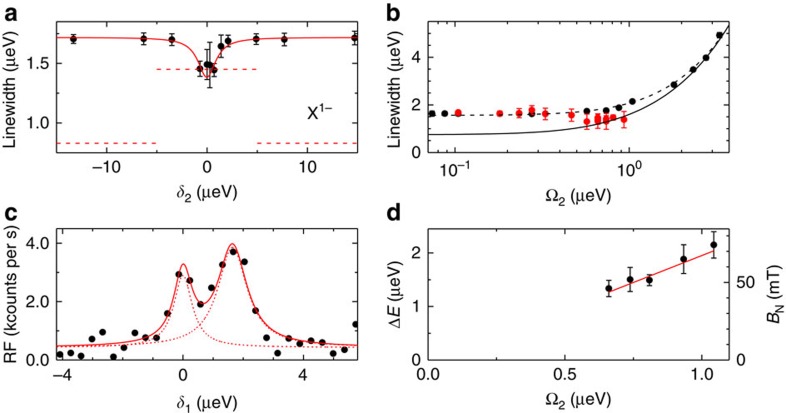
The two-laser experiment (*B*=0.0 mT, *T*=4.2 K) on X^1−^. (**a**) Optical linewidth measured with the probe laser (Ω_1_=0.23 μeV) versus detuning of the pump laser *δ*_2_ for Ω_2_=0.80 μeV. The dashed lines show the ideal case (zero inhomogeneous broadening) in two limits, Ω=Ω_1_ and Ω=Ω_1_+Ω_2_, appropriate for large *δ*_2_ and *δ*_2_=0.0 μeV, respectively, the difference arising from power broadening. (**b**) Optical linewidth in one-laser experiment (black points) versus Ω with fit to two-level model (*γ*=0.65 μeV (dashed line), 0 (solid line)). The optical linewidth in two-laser experiment (Ω_1_=0.23 μeV, *δ*_2_=0) versus Ω_2_ (red points). (**c**) Probe spectrum with Ω_1_=0.23 μeV, Ω_2_=0.80 μeV and *δ*_2_=0.0 μeV (points) with a two Lorentzian fit (solid line, energy separation 1.6 μeV, linewidths 0.8±0.3, 1.2±0.3 μeV). (**e**) Splitting from **d** versus Ω_2_. The error bars represent the s.d. of several scans.

We address whether the spin noise reduction in the case of X^1−^ is sufficient to achieve transform-limited optical linewidths. The Ω dependence of 
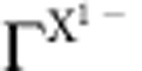
 can be described extremely well with the two-level result including an inhomogeneous broadening *γ* (Fig. 5b, Supplementary Note 7 and Supplementary Fig. 5). At low Ω, Γ is determined by Γ_0_ and *γ*; at higher Ω, Γ increases (power broadening) and *γ* becomes irrelevant. We can therefore determine the ideal limit (Γ versus Ω with *γ*=0) and below saturation, the inhomogeneous broadening is clearly significant (Fig. 5b). However, this relatively simple linewidth measurement is complex to interpret as the spin noise is a function of both Rabi energy and detuning. To simplify matters, we performed the experiment with two lasers. The concept is that the stronger, constant frequency pump laser (Ω_2_, *δ*_2_) determines the spin noise, and the weaker probe laser (Ω_1_, *δ*_1_) measures the optical linewidth. Figure 5a shows 
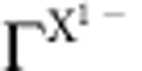
 measured by sweeping *δ*_1_ versus *δ*_2_ for Ω_1_=0.23, Ω_2_=0.80 μeV. For large *δ*_2_, the pump laser has no effect on Γ; power broadening is irrelevant and Γ is far from the transform limit. For small *δ*_2_ however, Γ decreases, despite the power broadening induced by Ω_2_. Taking into account power broadening, Γ reduces to the ideal limit. Figure 5b shows the results as Ω_2_ increases: for Ω/Γ_0_ >0.75, transform-limited optical linewidths are achieved (to within the random error of 10%). The spin noise reduction on driving X^1−^ with the pump laser is accompanied by a profound change in the probe spectrum: the optical resonance now splits into two resonances (Fig. 5c). The splitting reflects a static electron Zeeman splitting in the single-electron ground state, *B*_N_=58 mT in Fig. 5c, with *B*_N_ increasing with Ω_2_ (Fig. 5d, Supplementary Note 10 and Supplementary Fig. 8). Equivalently, even without an applied magnetic field^31^, a nuclear spin polarization is created by the optical coupling. This demonstrates that the laser locks the nuclear spins into an eigenstate of the Σ*I*_z_ operator. (We comment that significant nuclear spin polarizations can be achieved in an applied magnetic field^26^,^27^, for instance via ‘dragging' with resonant excitation^32^, but we find that the optical linewidths increase in this regime.)

## Discussion

The experiments reveal a remarkable dependence of the spin noise on charge. In the centre of the plateaus, resonant excitation of X^0^ enhances spin noise yet resonant optical excitation of X^1−^ suppresses spin noise. Concomitant with the different *B*_N,r.m.s._ values are the associated *B*_N_-correlation times, much shorter for X^0^ (5 μs) than for X^1−^ (100 μs)^21^,^33^. We note that the scanning frequency dependence (Fig. 2c) reveals a 100-μs noise correlation time for both X^0^ and X^1−^: at higher enough scanning frequencies, X^0^ is driven too briefly for any nuclear noise enhancement to be active. This points to the fact that the reduced correlation time and increased amplitude of the spin noise as measured on X^0^ is related to the constant optical driving. Fortunately, at a particular bias, the nuclear spin ‘shake-up' on driving X^0^ can be turned off and transform-limited linewidths can be achieved: the charge noise is too small to matter and the electron–hole exchange shields the exciton from the remaining nuclear spin noise.

Once the charge noise has been suppressed by using clean devices and the nuclear spin effects have been bypassed, the fidelity of the photons is limited by the phonons. The zero phonon line (ZPL) accounts for 95% of the emission^12^,^19^, a very high ratio for a solid-state emitter. The 5% non-ZPL photons can be filtered out without too much trouble but at the cost of a slight increase in shot noise. The phonon-related broadening of the ZPL is however very small at low temperature^9^,^12^. Once the device engineering described here has been combined with photonic mode engineering to boost the extraction efficiency^34^, there are excellent prospects for creating a fast and efficient source of indistinguishable photons using a semiconductor.

The mechanisms by which the nuclear spin noise respond to resonant optical excitation are unknown. For X^1−^, the data are compatible with a ‘narrowing' of the nuclear spin distribution, perhaps caused by continuous weak measurement via the narrowband laser^35^. The correlation time is compatible with the nuclear spin dipole–dipole interaction. For X^0^ it is unlikely that the standard electron spin–nuclear spin contact hyperfine interaction can offer an explanation; it is also unlikely that the bare dipole–dipole interaction can account for the short correlation time. One possibility is that the hole in the X^0^ is important. First, a hole has a complex hyperfine interaction, containing a term (*I*_+_*J*_z_+*I*−*J*_z_), exactly the structure required to shake-up the nuclear spins on creation of a hole (*I* is the nuclear spin and *J* the hole pseudo-spin)^36^. While the coefficient of this term is likely to be small, it can have significant consequences should the dark X^0^ state be occupied for times far exceeding the radiative lifetime^36^. A second possibility is that the hyperfine interaction renders the X^0^ sensitive to the nuclear spins by altering the phase effects that account for the electric field dependence of the fine structure splitting^37^. We hope that our results will stimulate a refinement in understanding of the exciton–nuclear spin interaction.

In conclusion, we report transform-limited optical linewidths from a single semiconductor quantum dot even when measured on second timescales on both X^0^ and X^1−^. Generally speaking, controlling spin noise is key to operating a quantum dot-based spin qubit^24^,^25^,^38^,^39^,^40^. The same factor turns out also to be a key feature in creating a quantum dot-based high-fidelity single-photon source.

## Methods

### The semiconductor quantum dot sample

The quantum dots are self-assembled using InGaAs in high-purity GaAs and are embedded between an n^+^ back contact (25-nm tunnel barrier) and a surface gate^17^,^21^ (Supplementary Methods and Supplementary Fig. 9). The gate voltage *V*_g_ determines the electron occupation via Coulomb blockade^41^.

### Resonance fluorescence

The quantum dot optical resonance is driven with a linearly polarized, resonant, continuous-wave laser (1 MHz linewidth) focused on to the sample surface. Reflected or scattered laser light is rejected with a dark-field technique using crossed linear polarizations for excitation and detection^42^. The laser excitation polarization is rotated by an angle of *π*/4 with respect to the neutral exciton's linear polarization axes.

Resonance fluorescence is detected with a silicon avalanche photodiode in photon-counting mode. The experiment is not shielded against the earth's magnetic field, thus *B*_min_ ∼50 μT. All the experiments were performed with the sample at 4.2 K. Γ is determined by sweeping the laser frequency through the resonance, integrating the counts, typically 100 ms per point.

## Additional information

**How to cite this article:** Kuhlmann, A. V. *et al*. Transform-limited single photons from a single quantum dot. *Nat. Commun.* 6:8204 doi: 10.1038/ncomms9204 (2015).

## Supplementary Material

Supplementary Information Supplementary Figures 1-9, Supplementary Notes 1-10, Supplementary Methods and Supplementary References.

## Figures and Tables

**Figure 1:**
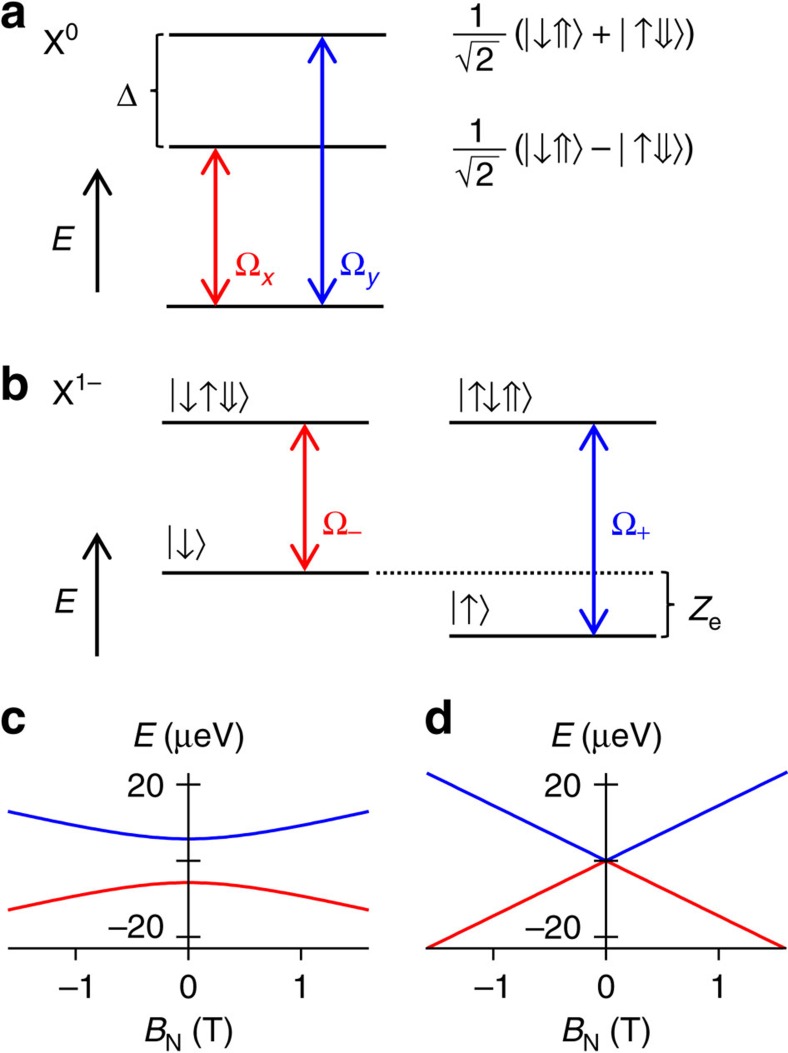
Neutral and charged excitons. (**a**) Energy levels of the neutral exciton X^0^ at zero magnetic field *B*=0, showing the fine structure splitting Δ. (**b**) Energy levels of the charged exciton X^1−^ in an Overhauser field *B*_N_, introducing an electron Zeeman splitting *Z*_e_. (**c**,**d**) X^0^, X^1−^ energy levels versus *B*_N_ with Δ=11.5 μeV and electron g-factor *g*=−0.5.
