# Supplementary material for: Transform-limited single photons from a single quantum dot
Source: Nat Commun. 2015 Sep 8;6:8204. doi: 10.1038/ncomms9204 (PMC4569856; doi:10.1038/ncomms9204)
Supplement: Supplementary Information — Supplementary Figures 1-9, Supplementary Notes 1-10, Supplementary Methods and Supplementary References. [file ncomms9204-s1.pdf]

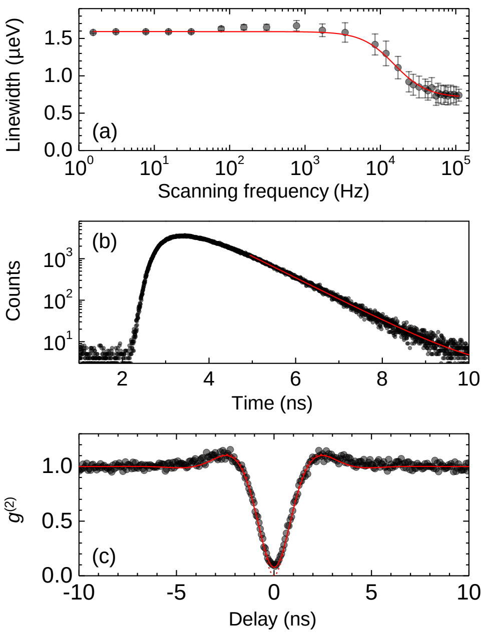

Supplementary Figure 1: Determination of the quantum dot radiative lifetime $\tau_{\rm r}$. (a) Linewidth as a function of scanning frequency, $\tau_{\rm r} = 880 \pm 50$ ps; (b) decay curve following pulsed non-resonant excitation, $\tau_{\rm r} = 850 \pm 50$ ps; (c) an intensity correlation $g^{(2)}$, $\tau = 820 \pm 50$ ps. (a)-(c) are all recorded on $\rm X^{1-}$ on the same quantum dot. See Supplementary Note 1 for further discussion.

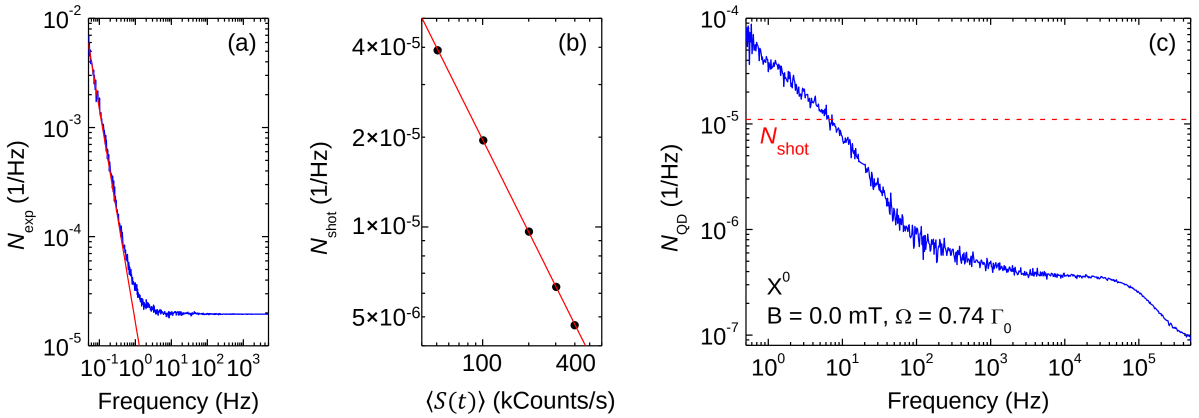

Supplementary Figure 2: Noise spectrum of the experiment. (a) Intensity fluctuations of the laser light in the setup cause a $1/f^2$-behaviour of $N_{\mathrm{exp}}(f)$ at low frequencies (exponent of red fit -1.96). For $f > 10$ Hz the spectrum is dominated by shot noise, thus, the spectrum is flat. The average count rate of the detected laser light is 101 kCounts/s in this particular experiment. (b) Shot noise. Noise spectra of the experiment alone were recorded at different laser light count rates to extract the dependence of the shot noise on the count rate. A proportionality of the shot noise to $\langle S(t) \rangle^{-1}$ is verified (exponent of red fit -1.03). (c) Quantum dot noise spectrum. The noise of the experiment is typically larger than the noise of the QD. The shot noise (red dashed line) typically equals $N_{\mathrm{QD}}(f)$ at low frequencies ($f \sim 10$ Hz), and exceeds $N_{\mathrm{QD}}(f)$ at higher frequencies. The RF count rate is 176 kCounts/s in this particular experiment. Data from QD2. See Supplementary Note 2 for further discussion.

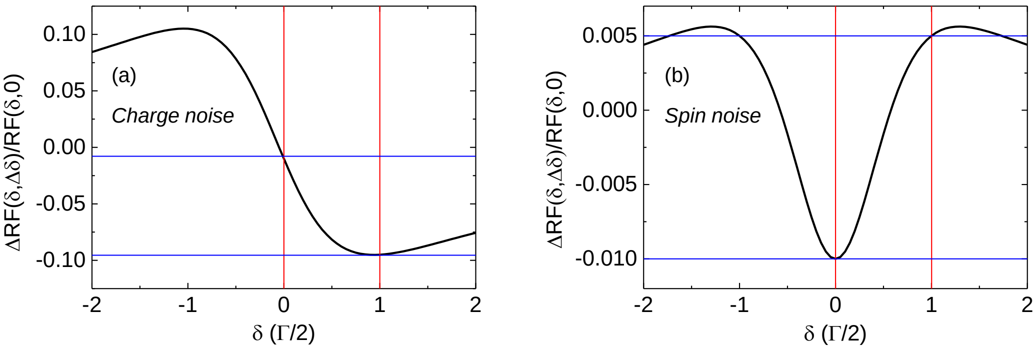

Supplementary Figure 3: Noise sensitivity dependence on detuning. Sensitivity for charge noise (a) and spin noise (b) for both X^0 (two lasers with frequency splitting equal to the fine structure) and X^{1-} (one laser). The relative change in the RF caused by an energy fluctuation of $\Gamma_0/20$ is shown as a function of detuning δ. The blue lines indicate the noise sensitivity for $\delta = 0$ and $\delta = \Gamma/2$. See Supplementary Note 4 for further discussion.

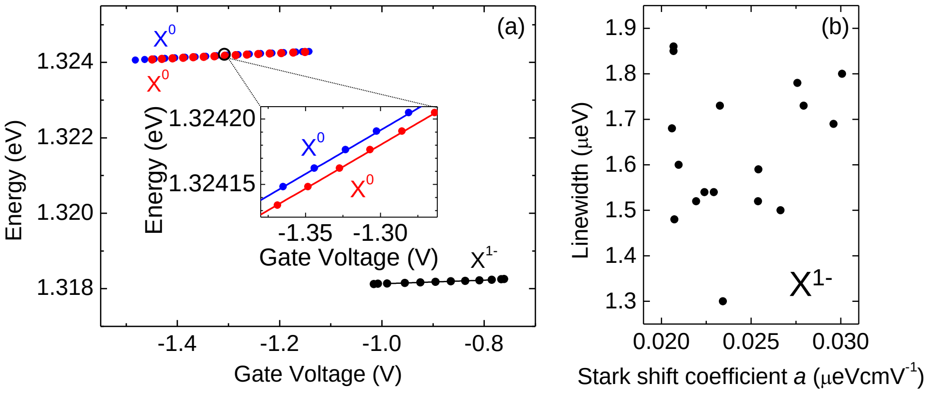

Supplementary Figure 4: The dc Stark effect. (a) Exciton energy voltage plateaus to determine the Stark shift coefficients of the neutral exciton X^0 and the trion X^{1-}. Inset shows a zoom in of X^0 revealing the fine structure splitting $\Delta = 11.5\,\mu$eV. Data from QD1. (b) Linewidth versus Stark shift. Statistics on X^{1-} of 17 QDs from the same wafer with a spread in Stark shift of up to 50% demonstrate no significant correlation between linewidth and Stark shift. See Supplementary Note 6 for further discussion.

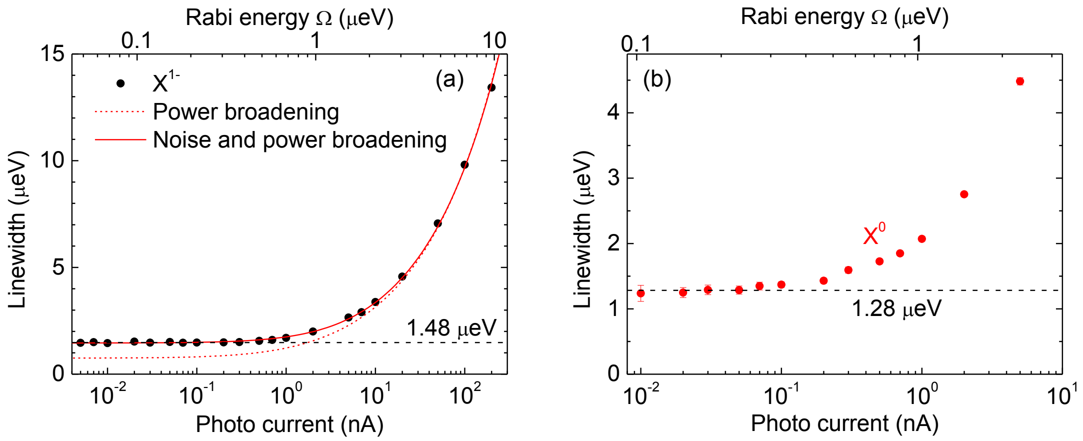

Supplementary Figure 5: Power broadening. Linewidth power dependence for (a) X^{1-} and (b) X^0. The 2-level model with (solid red lines) and without (dashed red lines) an inhomogeneous broadening ($\gamma = 0.56\,\mu$eV) is fitted to the X^{1-} data. The transform-limit Γ_0 is $0.75\,\mu$eV for X^{1-} and $0.92\,\mu$eV for X^0. Data from QD1. See Supplementary Note 7 for further discussion.

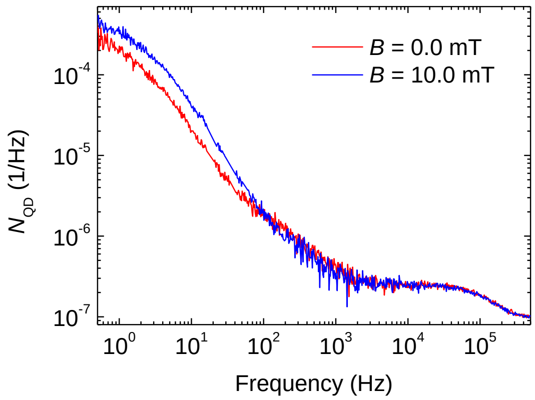

Supplementary Figure 6: Noise measurements on X^0 at $\Omega = 0.45\ \mu$eV with and without a small magnetic field. The small difference in charge noise is due to a different charging configuration of the device (see history effects in Ref. [1]). Data from QD1. See Supplementary Note 8 for further discussion.

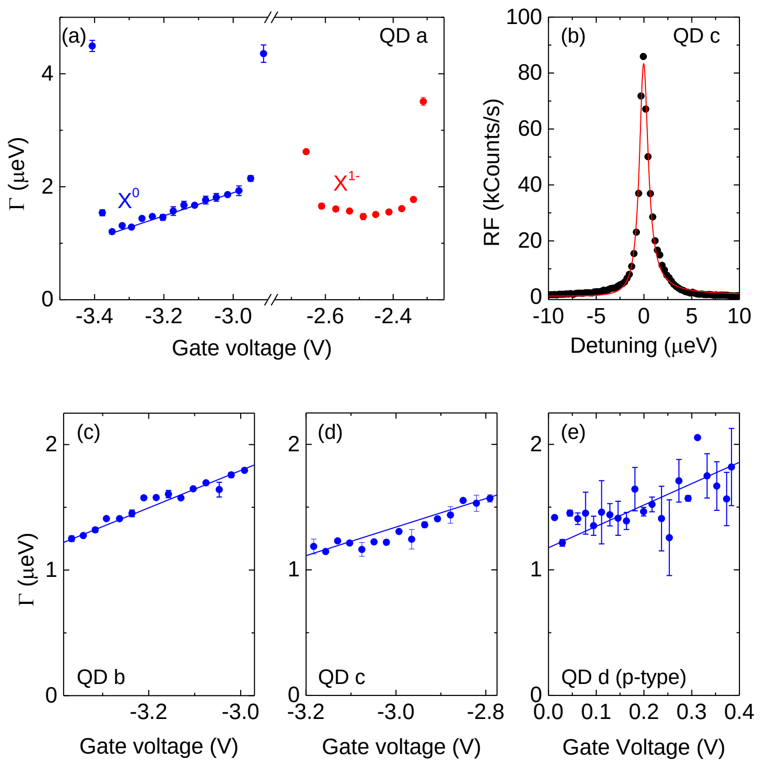

Supplementary Figure 7: Statistics on quantum dots. (a) X^0 and X^{1-} optical linewidths measured for different gate voltages by sweeping the laser frequency through the resonance and integrating for 100 ms per point. For X^0, the smallest optical linewidth is observed at the negative end of the Coulomb blockade; for X^{1-}, at the centre of the plateau. (b) X^0 spectrum with $\Gamma = 1.13$ μeV. Taking power broadening into account the ideal limit is $\Gamma_0 = 1.14 \pm 0.15$ μeV. The slight asymmetry in the lineshape results from an imperfect laser suppression in this particular case. (c)-(e) X^0 optical linewidth versus gate voltage for three additional quantum dots. All the QDs exhibit a sweet-spot at the negative end of the Coulomb blockade plateau. See Supplementary Note 9 for further discussion.

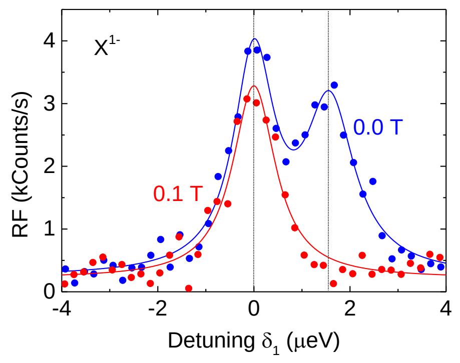

Supplementary Figure 8: Two-laser experiment performed with identical parameters ($\Omega_1 = 0.15$ μeV, $\Omega_2 = 0.5$ μeV) on X^{1-} with and without a magnetic field. Data from QD1. See Supplementary Note 10 for further discussion.

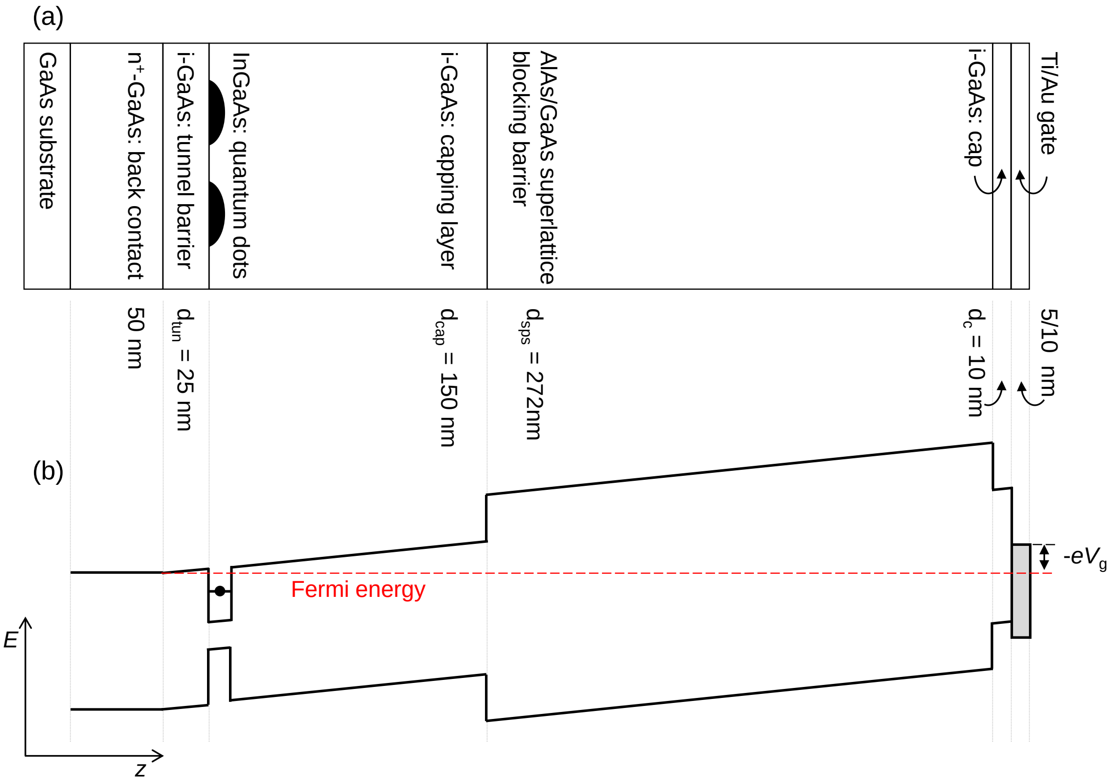

Supplementary Figure 9: The quantum dot sample. (a) Sample layer structure and the corresponding (b) energy band diagram. The Fermi energy is pinned to the conduction band edge of the back contact. The figures are to scale with respect to length. See Supplementary Methods for further discussion.

Supplementary Note 1: Radiative lifetime

To compare the optical linewidth to the ideal case it is clearly necessary to know the transform-limit, equivalently the radiative lifetime τ_r. We measure the radiative lifetime in three different ways, Supplementary Fig. 1. First, we measure the linewidth as a function of scanning frequency. The linewidth is a constant and then decreases between scanning frequencies of ~ 5 kHz and ~ 50 kHz to another constant value [1]. Given the known absence of upper level decoherence under these conditions (low temperature, high quality material, weak resonant excitation) this constant value at high scanning frequencies corresponds to the transform-limit. Secondly, we measure the radiative lifetime by recording a decay curve following excitation with a non-resonant pulse. Analysis of the decay, taking account the timing jitter of the detector, reveals the radiative lifetime. Finally, we record an intensity correlation function $g^{(2)}$ with a Hanbury Brown-Twiss setup, fitting the results to the behaviour of a resonantly-driven two-level atom convoluted with the jitter function of the detectors. We find that all three results give the same value for the radiative lifetime to within the measurement uncertainty of $\sim 5\%$.

From quantum dot to quantum dot, there is a systematic difference between X^0 and X^{1-}: the radiative decay rate is larger for X^0 than for X^{1-} (Fig. 2c of the main article). The difference however from quantum dot to quantum dot is small, just $\sim 5\%$.

Supplementary Note 2: Quantum dot noise spectrum

To determine the QD noise spectrum the arrival time of each photon is recorded over the entire measurement time T. Post measurement, a binning time $t_{\rm bin}$ is selected, typically $1\,\mu$s. The number of counts in each time bin is $S(t)$, the average number of counts per bin $\langle S(t)\rangle$. The fast Fourier transform of the normalized RF signal $S(t)/\langle S(t)\rangle$ is calculated to yield a spectrum of the noise power $N_{\rm RF}(f)$, specifically

$$N_{\rm RF}(f) = |{\rm FFT}\,[S(t)/\langle S(t)\rangle]|^2\,(t_{\rm bin})^2/T. \tag{1}$$

$N_{\rm RF}(f)$ has the same spectrum independent of the choice of $t_{\rm bin}$ and T: smaller values of $t_{\rm bin}$ allow $N_{\rm RF}(f)$ to be determined to higher values of frequency f; larger values of T allow $N_{\rm RF}(f)$ to be determined with higher resolution. The high frequency limit of our experiment is only limited by the photon flux.

All Fourier transforms are normalized [2] such that the integral of the noise power $N_x(f)$ over all positive frequencies equals the variance of the fluctuations δx,

$$\langle(\delta x)^2\rangle = \int_0^\infty df N_x(f). \tag{2}$$

To record a noise spectrum of the experiment alone, the QD is detuned by > 100 linewidths relative to the laser and one polarizer is rotated by a small angle to open slightly the detection channel for reflected laser light, choosing the rotation so that the detected laser light gives a count rate similar to the QD RF. A noise spectrum of the reflected laser light (Supplementary Fig. 2 (a)) is recorded using exactly the routine used to analyse the RF, yielding $N_{\rm exp}(f)$. $N_{\rm exp}(f)$ has a $1/f^2$-behaviour at low frequencies arising from intensity fluctuations in the setup. For $f > 10$ Hz, $N_{\rm exp}(f)$ has a completely f-independent spectrum, $N_{\rm exp} \sim 10^{-5}$ Hz^{-1}: this is the shot noise $N_{\rm shot}$. The noise of the experiment is typically larger than the noise of the QD $N_{\rm QD}(f)$. The shot noise is proportional to $\langle S(t)\rangle^{-1}$ (Supplementary Fig. 2 (b)) and not to $\langle S(t)\rangle^{1/2}$ due to the normalization of $S(t)$ by $\langle S(t)\rangle$ in the calculation of the spectrum. $N_{\rm shot}$ is comparable to $N_{\rm QD}(f)$ at low frequencies ($f \sim 10$ Hz), and exceeds $N_{\rm QD}(f)$ at higher frequencies, Supplementary Fig. 2 (c).

The noise spectrum of the QD alone is then determined using

$$N_{\rm QD}(f) = N_{\rm RF}(f) - N_{\rm exp}(f). \tag{3}$$

Correction of $N_{\rm RF}(f)$ with $N_{\rm exp}(f)$ where $N_{\rm RF}(f)$ and $N_{\rm exp}(f)$ are not measured simultaneously is successful on account of the high stability of the setup. Furthermore, no spectral resonances in $N_{\rm QD}(f)$ have been discovered. We present here $N_{\rm QD}(f)$ after averaging at each f over a frequency range Δf to yield equidistant data points on a logarithmic scale. This entire procedure enables us to discern $N_{\rm QD}(f)$ down to values of 10^{-7} Hz^{-1} for $T = 2$ hours.

Supplementary Note 3: Noise spectra modelling

Our previous experiments [1] demonstrate that the spectrum of the noise in the RF is dominated by charge noise at low frequency, spin noise at high frequency. The noise sensor, the RF from a single quantum dot, has a trivial dependence on the fluctuating electric $F(t)$ and magnetic fields $B_N(t)$ only for small fluctuations in the detunings around particular values of detuning δ. Monte Carlo simulations allow us to determine both the electric field and magnetic field noise accurately by describing the response of the sensor for all δ, treating charge noise and spin noise on an equal footing.

The basic approach is to calculate $F(t)$ and $B_N(t)$, in each case from an ensemble of independent, but identical, 2-level fluctuators using a Monte Carlo method; to calculate the RF signal $S(t)$ from $F(t)$ and $B_N(t)$; and to compute the noise $N(f)$ from $S(t)$ using exactly the same routine as for the experiments (but without the correction for extrinsic noise of course). Here, we discuss the spin noise modelling of the neutral exciton X^0 used to extract the root-mean-square (rms) values of the magnetic field $B_{N,\mathrm{rms}}$ in Supplementary Fig. 4 (c) of the main article. The modelling of charge noise is explained in detail elsewhere [1].

For X^0, the RF depends on the electric and magnetic fields according to

$$S(t) = \frac{\left(\frac{\Gamma_0}{2}\right)^2}{\left(aF(t) + \delta_0(t) + \delta\right)^2 + \left(\frac{\Gamma_0}{2}\right)^2}, \quad \delta_0(t) = \pm\frac{1}{2}\sqrt{\Delta^2 + \delta_1(t)^2}, \quad \delta_1(t) = \frac{1}{2} g\mu_B B_N(t), \tag{4}$$

where a is the dc Stark coefficient, g the electron g-factor and Δ the fine structure splitting. For the blue Zeeman branch $\delta_0(t)$ is positive, for the red one negative, respectively.

An ensemble of identical 2-level fluctuators fully describes spin noise, Fig. 4 (a) of the main article.

A 2-level fluctuator occupies either state 0 with lifetime τ_0 or state 1 with lifetime τ_1. The probability p of being, at any time, in state 1 is $\tau_1/(\tau_0+\tau_1)$; the probability of being in state 0 is $\tau_0/(\tau_0+\tau_1)$. The configuration $C(t)$ of a 2-level fluctuator, either 0 or 1, is determined by the probabilities of a $0 \to 1$ transition [3],

$$p_{0\to1}(\delta t) = 1 - \frac{1}{\tau_0+\tau_1}\left[\tau_1 \exp\left(-\left(\frac{1}{\tau_0}+\frac{1}{\tau_1}\right)\delta t\right) + \tau_0\right] \tag{5}$$

and a $1 \to 0$ transition,

$$p_{1\to0}(\delta t) = 1 - \frac{1}{\tau_0+\tau_1}\left[\tau_0 \exp\left(-\left(\frac{1}{\tau_0}+\frac{1}{\tau_1}\right)\delta t\right) + \tau_1\right] \tag{6}$$

where δt denotes the time over which the system evolves. The power spectrum of a 2-level fluctuator $S(\omega)$ is Lorentzian [3],

$$S(\omega) = \frac{1}{\pi}\frac{\tau_0\tau_1}{(\tau_0+\tau_1)^2}\frac{1/T}{\omega^2+(1/T)^2}, \quad 1/T = 1/\tau_0 + 1/\tau_1. \tag{7}$$

The calculation of the time trace of the magnetic field $B_N(t)$ is simplified, such that each nucleus is treated as a two-level fluctuator, with equal $0 \to 1$, $1 \to 0$ transition rates, $1/\tau$. At $t = 0$, each nucleus is initialized by a random number generator giving a configuration of nuclear spins $C(0)$. At a later time, δt, $C(\delta t)$ is calculated from $C(0)$ again with a random number generator using the probabilities $p_{1\to 0}(\delta t)$ and $p_{0\to 1}(\delta t)$ from the theory of a two-level fluctuator. The nuclei are treated independently.

The nuclear magnetic field, the so-called Overhauser field B_N, is given by [4]

$$B_N = \frac{v_0}{g\mu_B} \sum_{i=1}^{N} A_i \left|\psi(\mathbf{r}_i)\right|^2 I_i \tag{8}$$

where v_0 is the atomic volume, A_i the hyperfine interaction constant, $\mathbf{r}_i$ is the position of the nuclei i with spin I_i, and $\psi(\mathbf{r})$ is the normalized electron envelope function. By using an average hyperfine constant [5] $A = 90\,\mu$eV and approximating the electron envelope function $\psi(\mathbf{r})$ by a top hat, Eq. (8) simplifies to

$$B_N = \frac{A}{g\mu_B N_{\text{eff}}} \sum_{i=1}^{N_{\text{eff}}} I_i. \tag{9}$$

N_{eff} denotes the number of nuclear spins inside the top hat envelope function.

Regarding the dimensionality of B_N, a 1D model for the nuclear spins is appropriate for X^0. The isotropic part of the electron-hole exchange interaction "protects" the X^0 from the in-plane fluctuations of the nuclear magnetic field. Specifically, the z-component of the Overhauser field enters along the diagonals of the exchange/Zeeman Hamiltonian [6] in the $|\Uparrow\downarrow\rangle$, $|\Downarrow\uparrow\rangle$, $|\Uparrow\uparrow\rangle$, $|\Downarrow\downarrow\rangle$ basis and results in the dispersion of Eq. 4. The in-plane components of the Overhauser field couple $|\Uparrow\downarrow\rangle \leftrightarrow |\Uparrow\uparrow\rangle$ and $|\Downarrow\uparrow\rangle \leftrightarrow |\Downarrow\downarrow\rangle$ but these states are split by the dark-bright splitting, 100s of μeV, determined by the isotropic part of the exchange interaction. As a result the dependence of the exciton energy on the in-plane fields is negligible.

We assume that each nuclear spin I can be represented by a spin-$\frac{1}{2}$, a 2-level fluctuator. To account for an underestimate of the hyperfine interaction (the real spins are larger than $\frac{1}{2}$) the Overhauser field is enhanced via a reduction in the total number of nuclei, $N \to N_{\text{eff}}$. Equivalently, we could work with a higher N_{eff} and larger A. The model represents a phenomenological way to create $B_N(t)$ which mimics the experiment. $B_N(t)$ is unique, the route to $B_N(t)$ is not.

There are two independent parameters that control spin noise in the simulation: the correlation time τ and the rms field $B_{N,\text{rms}}$. For the simulation shown in Fig. 4 (a) of the main article $A = 90\,\mu$eV, $N_{\text{eff}} = 178$, corresponding to $B_{N,\text{rms}} = 116\,\text{mT}$, and $\tau = 6.0\,\mu$s were used. The noise spectra at higher Rabi energies were fitted by decreasing N_{eff} (increasing $B_{N,\text{rms}}$) and the same τ.

Supplementary Note 4: Charge noise and spin noise sensitivity dependence on laser detuning

The sensitivity in the RF to charge noise and spin noise depends on the laser detuning δ, Supplementary Fig. 3. For X^{1-}, only one laser is required to distinguish charge noise and spin noise yet two lasers with frequencies separated by the fine structure splitting are required for X^0. Both charge noise (Supplementary Fig. 3 (a)) and spin noise (Supplementary Fig. 3 (b)) exhibit the same detuning dependence for X^{1-} (one laser) and X^0 (two lasers). On detuning the laser/both lasers (X^{1-}/X^0) from $\delta = 0$ to $\delta = \Gamma/2$, the sensitivity to charge noise changes from second order to first order yet the sensitivity to spin noise decreases by a factor $\sim$2.

Supplementary Note 5: Effect of charge noise on the linewidth

The quantum dot noise spectrum $N_{\rm QD}(f)$ allows us to set an upper limit of the linewidth broadening $\gamma_{\rm c}$ due to charge noise. The energy jitter due to charge fluctuations is less than the linewidth such that the change in RF is related quadratically to the detuning for fluctuations around $\delta = 0$. This quadratic approximation overestimates the effect of charge fluctuations on the linewidth. The variance of the quantum dot RF noise, $\sigma^2_{\rm QD,c}$, is related to an integral of the noise curve. Integrating over the bandwidth of charge noise after subtracting spin noise,

$$\gamma_{\rm c} = \frac{\Gamma}{2}\left(\sigma^2_{\rm QD,c}/3\right)^{1/4}. \tag{10}$$

The charge noise has a $1/f$-like component and a Lorentzian component. We integrate both from 0.1 Hz to 1 GHz. Applying this concept to the X^{1-} noise spectrum of Fig. 4 (b) of the main article, with $\Gamma = 1.48\,\mu$eV this predicts $\gamma_{\rm c} < 0.05\,\mu$eV.

Supplementary Note 6: The dc Stark effect

The Stark shift is determined by recording the resonance position in V_g for many laser frequencies, the laser frequency measured in each case with an ultra-precise wavemeter. The Stark shift is linear in ΔF for the small windows of V_g used here, Supplementary Fig. 4 (a). The neutral exciton X^0 has a larger Stark shift ($a = 0.0306\,\mu\mathrm{eVcm/V}$) than the charged exciton X^{1-} ($a = 0.0219\,\mu\mathrm{eVcmV}^{-1}$) and thus it is more sensitive to charge noise. X^{1-} has a larger linewidth ($\Gamma = 1.48$ μeV) compared to X^0 ($\Gamma = 1.28$ μeV) despite the smaller Stark shift. Also, experiments on several QDs reveal no dependence of the linewidth on the Stark shift coefficient, Supplementary Fig. 4 (b). The Stark shift varies from quantum dot to quantum dot by up to 50% without a correlated change in linewidth. Both these facts support the dominant influence of spin noise and not charge noise on the X^0 and X^{1-} linewidths.

Supplementary Note 7: Power broadening

The linewidth of the optical resonance increases with increasing resonant excitation power, Supplementary Fig. 5. The additional contribution to the linewidth is known as *power broadening*, described for an ideal 2-level system by [7]

$$\Gamma(\Omega) = \sqrt{\Gamma_0^2 + 2\Omega^2} + \gamma, \quad \Gamma_0 = \hbar/\tau_R \tag{11}$$

with Rabi energy Ω and radiative lifetime τ_R. An inhomogeneous broadening is included with the term γ.

For X^{1-}, the 2-level model with constant γ describes the data very well, Supplementary Fig. 5 (a). The inhomogeneous broadening γ is constant at low power, decreasing at high power but only when power broadening dominates, such that a constant γ allows the experimental data to be described very well (see main article). By fitting the 2-level model to the data a resonant excitation power measured by a photo diode beneath the sample can be converted to a Rabi energy, Supplementary Fig. 5 (a).

Conversely for X^0, the 2-level model with constant γ does not describe the data well. The inhomogeneous broadening is strongly power dependent: γ increases significantly with increasing resonant excitation power (see main article).

A phonon-induced dephasing process as observed at very high Rabi couplings [8] and in pulsed experiments [9] is negligible at these Rabi couplings.

Supplementary Note 8: Effect of a small magnetic field on the nuclear spin dynamics

A small magnetic field of $B = 10.0$ mT was applied to measure the Ω-dependence of $N_{\mathrm{QD}}(f)$, Fig. 4 of the main article. As a result, the sensitivity of the charged exciton to spin noise is increased [1]. The nuclear spin dynamics are however not strongly changed by such a small magnetic field. Noise measurements on X^0 with and without a small magnetic field of 10.0 mT demonstrate an unchanged spin noise level, Supplementary Fig. 6.

Supplementary Note 9: Statistics on quantum dots

We investigated quantum dots in the same sample, in other samples from the same wafer and from samples from other wafers of a similar but non-identical design to question the robustness of the results.

For X^0, all the quantum dots exhibit the "sweet-spot" at the negative end of the Coulomb blockade plateau, Supplementary Fig. 7, where the optical linewidth exhibits a minimum. The minimum optical linewidth equals the transform-limit (to within the random error of 10%). A very striking example is the observation of the sweet-spot on a p-type field-effect device, Supplementary Fig. 7 (e). These results, recorded in detail on 10 quantum dots, therefore establish the sweet-spot as a robust phenomenon allowing transform-limited optical linewidths to be observed by simply applying the correct bias to the device.

For X^{1-}, the smallest optical linewidth is observed at the centre of the Coulomb blockade plateau, Supplementary Fig. 7 (a). At the edges of the plateau the optical linewidth rises rapidly due to electron co-tunneling [10]. Transform-limited optical linewidths can be achieved by choosing a gate voltage somewhere close to the centre of the plateau and then suppressing the spin noise with the resonant laser. This too, as for X^0, is a robust phenomenon.

Supplementary Note 10: Relationship to Autler-Townes splitting

In a two-laser experiment at zero magnetic field with a resonant pump laser the optical resonance of the charged exciton splits into two resonances. The splitting reflects a static electron Zeeman splitting in the single electron ground-state and not an Autler-Townes splitting [11]. We can rule out an Autler-Townes splitting as first, the splitting is not given by the Rabi energy Ω as is the case for an Autler-Townes splitting, and secondly, we do not observe an optically-induced splitting when the X^{1-} resonance is pulled apart in a small magnetic field, Supplementary Fig. 8.

Supplementary Methods

A quantum dot (QD) sample grown by molecular beam epitaxy is used to probe the optical linewidth of single photons from a single QD. All the data presented in the main article were measured on three QDs from different samples: QD1 from sample A (Fig.s 1, 2 (a)-(b), 4 and 5), QD2 from sample B (Fig. 2 (c)) and QD3 from sample C (Fig. 3).

The self-assembled QDs are embedded in a Schottky diode [12, 13] as shown in Supplementary Fig. 9 (a). The layer sequence is:

1. *back contact*
   50 nm n^+-GaAs, doping level $\sim 1.7 \times 10^{18}\,\mathrm{cm}^{-3}$

2. *tunnelling barrier*
   25 nm i-GaAs

3. *active layer*
   InGaAs QDs (diameter $\sim$ 20 nm, height $\sim$ 5 nm) with centre wavelength 950 nm

4. *capping layer*
   150 nm i-GaAs

5. *blocking barrier*
   68 periods AlAs/GaAs 3 nm/1 nm

6. *cap*
   10 nm i-GaAs

7. *Schottky gate*
   sample A 5 nm/10 nm Ti/Au
   sample B and C 3 nm/7 nm Ti/Au.

The samples only differ in the gate thickness: they are fabricated from the same wafer.

The background doping of as-grown GaAs is p $\sim 10^{13}\,\mathrm{cm}^{-3}$; two-dimensional electron gases grown under similar conditions have mobilities $> 10^6\,\mathrm{cm}^2\,\mathrm{V}^{-1}\mathrm{s}^{-1}$.

The number of electrons confined to the QD can be precisely controlled by the gate voltage V_g as illustrated in Supplementary Fig. 9 (b). A change of gate voltage yields a change of the QD's local potential ϕ by

$$\Delta\phi = \frac{\Delta V_\mathrm{g}}{\lambda} \tag{12}$$

where $\lambda = 18.3$ denotes the sample's lever arm, defined as the ratio of back contact to gate distance d and tunnel barrier thickness. The exciton energy E is detuned with respect to the constant laser frequency by exploiting the dc Stark effect,

$$\Delta E = a\Delta F, \quad \Delta F = \frac{\Delta V_{\mathrm{g}}}{d} \tag{13}$$

with Stark shift coefficient a and electric field F.

Supplementary references

[1] Kuhlmann, A. V. *et al.* Charge Noise and Spin Noise in a Semiconductor Quantum Device. *Nat. Phys.* **9**, 570–575 (2013).

[2] Kogan, S. *Electronic Noise and Fluctuations in Solids* (Cambridge University Press, London, 1996).

[3] Machlup, S. Noise in Semiconductors: Spectrum of a Two-Parameter Random Signal. *J. Appl. Phys.* **25**, 341–343 (1954).

[4] Urbaszek, B. *et al.* Nuclear spin physics in quantum dots: An optical investigation. *Rev. Mod. Phys.* **85**, 79–133 (2013).

[5] Kloeffel, C. *et al.* Controlling the Interaction of Electron and Nuclear Spins in a Tunnel-Coupled Quantum Dot. *Phys. Rev. Lett.* **106**, 046802 (2011).

[6] Bayer, M. *et al.* Fine Structure of Neutral and Charged Excitons in Self-assembled In(Ga)As/(Al)GaAs Quantum Dots. *Phys. Rev. B* **65**, 195315 (2002).

[7] Loudon, R. *The Quantum Theory of Light* (Oxford University Press, 2010).

[8] Ulrich, S. M. *et al.* Dephasing of Triplet-Sideband Optical Emission of a Resonantly Driven $\mathrm{InAs/GaAs}$ Quantum Dot inside a Microcavity. *Phys. Rev. Lett.* **106**, 247402 (2011).

[9] Ramsay, A. J. *et al.* Damping of Exciton Rabi Rotations by Acoustic Phonons in Optically Excited InGaAs/GaAs Quantum Dots. *Phys. Rev. Lett.* **104**, 017402 (2010).

[10] Smith, J. M. *et al.* Voltage Control of the Spin Dynamics of an Exciton in a Semiconductor Quantum Dot. *Phys. Rev. Lett.* **94**, 197402 (2005).

[11] Gerardot, B. D. *et al.* Dressed excitonic states and quantum interference in a three-level quantum dot ladder system. *New Journal of Physics* **11**, 013028 (2009).

[12] Drexler, H., Leonard, D., Hansen, W., Kotthaus, J. P. & Petroff, P. M. Spectroscopy of Quantum Levels in Charge-Tunable InGaAs Quantum Dots. *Phys. Rev. Lett.* **73**, 2252–2255 (1994).

[13] Warburton, R. J. *et al.* Optical Emission from a Charge-Tunable Quantum Ring. *Nature (London)* **405**, 926–929 (2000).

[14] Kuhlmann, A. V. *et al.* A Dark-Field Microscope for Background-Free Detection of Resonance Fluorescence from Single Semiconductor Quantum Dots Operating in a Set-and-Forget Mode. *Rev. Sci. Instrum.* **84**, 073905 (2013).
